# Rapidly progressive sporadic Creutzfeldt-Jakob disease: isolated Heidenhain variant or a combination with PRES?

**DOI:** 10.1590/0004-282X-ANP-2020-0428

**Published:** 2021-04-23

**Authors:** Pedro Henrique Almeida FRAIMAN, Carolina Militão TEIXEIRA, Juliano César Dantas DE OLIVEIRA, Thadeu Alexandre Paulino DE SOUSA, Manuel MOREIRA-NETO, Clecio de Oliveira GODEIRO-JUNIOR

**Affiliations:** 1 Universidade Federal do Rio Grande do Norte, Hospital Universitário Onofre Lopes, Divisão de Neurologia, Natal RN, Brazil. Universidade Federal do Rio Grande do Norte Universidade Federal do Rio Grande do Norte Hospital Universitário Onofre Lopes Divisão de Neurologia Natal RN Brazil; 2 Casa de Saúde São Lucas, Divisão de Radiologia, Natal RN, Brazil. Casa de Saúde São Lucas Divisão de Radiologia Natal RN Brazil; 3 Universidade Federal do Rio Grande do Norte, Hospital Universitário Onofre Lopes, Divisão de Radiologia, Natal RN, Brazil. Universidade Federal do Rio Grande do Norte Universidade Federal do Rio Grande do Norte Hospital Universitário Onofre Lopes Divisão de Radiologia Natal RN Brazil

A 70-year-old man presented with rapidly progressive cognitive impairment with ataxia and myoclonus. Visual agnosia was noticed after blood pressure oscillations, during immunoglobulin therapy for suspected autoimmune encephalitis. Brain magnetic resonanbce imaging scans before and after visual agnosia are presented in Figure 1. Electroencephalogram disclosed periodic sharp wave complex, and cerebrospinal fluid was positive for 14-3-3 protein. The final diagnosis was probable Creutzfeldt-Jakob disease (CJD) associated with posterior reversible encephalopathy syndrome (PRES). Blood pressure and immunoglobulin therapy may explain PRES^1^. It seems that some types of CJD prion proteins could induce the activation of microglia and dysfunction of vasoconstrictors upregulation, leading to vasospasm, ischemia and PRES^1^^,^^2^

**Figure 1.**
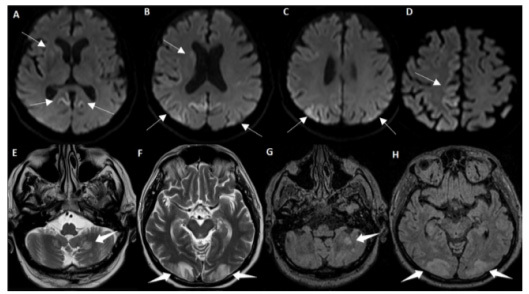
Magnetic resonance imaging features of sporadic Creutzfeld-Jakob disease and posterior reversible encephalopathy syndrome. (A, B, C and D) Initial diffusion-weighted images revealing bilateral hyperintensities in parietal areas, mostly on the right, and hyperintensities in cingulate gyrus. The same findings apply albeit to a lesser extent in the right basal ganglia (caudate and putamen, white arrows). (E, F, G and H) T2WI and T2-fluid-attenuated inversion-recovery reveals new cortico-subcortical hyperintensities in parietal and occipital areas and cerebellum, compatible with vasogenic edema, highly suggestive of PRES (thick white arrows). Previously described alterations remain the same. T1W1 does not reveal contrast enhancement.

## References

[1] 1. Dirzius E, Balnyte R, Steibliene V, Gleizniene R, Gudinaviciene I, Radziunas A, et al. Sporadic Creutzfeldt-Jakob disease with unusual initial presentation as posterior reversible encephalopathy syndrome: a case report. BMC Neurol. 2016 Nov;16(1):234. https://doi.org/10.1186/s12883-016-0751-8 10.1186/s12883-016-0751-8 PMC5120446 27876002

[2] 2. Freitas CS, Pinheiro MGM, Fonte EJD, Hazin AN, Smid J, Barbosa BJAP. Posterior cortical ribboning in the Heidenhain variant of Creutzfeldt-Jakob Disease. Arq Neuro-Psiquiatr. 2020 Apr;78(4):241. https://doi.org/10.1590/0004-282x20190176 10.1590/0004-282X20190176 32321050

